# SubcellulaRVis: a web-based tool to simplify and visualise subcellular compartment enrichment

**DOI:** 10.1093/nar/gkac336

**Published:** 2022-05-10

**Authors:** Joanne Watson, Michael Smith, Chiara Francavilla, Jean-Marc Schwartz

**Affiliations:** Division of Evolution, Infection & Genomic Sciences, School of Biological Sciences, Faculty of Biology, Medicine & Health, University of Manchester, Manchester M13 9PT, UK; Division of Molecular & Cell Biology, School of Biological Sciences, Faculty of Biology, Medicine & Health, University of Manchester, Manchester M13 9PT, UK; Division of Molecular & Cell Biology, School of Biological Sciences, Faculty of Biology, Medicine & Health, University of Manchester, Manchester M13 9PT, UK; Division of Molecular & Cell Biology, School of Biological Sciences, Faculty of Biology, Medicine & Health, University of Manchester, Manchester M13 9PT, UK; Manchester Breast Centre, Manchester Cancer Research Centre, The University of Manchester, M139PT, Manchester, UK; Division of Evolution, Infection & Genomic Sciences, School of Biological Sciences, Faculty of Biology, Medicine & Health, University of Manchester, Manchester M13 9PT, UK

## Abstract

Cells contain intracellular compartments, including membrane-bound organelles and the nucleus, and are surrounded by a plasma membrane. Proteins are localised to one or more of these cellular compartments; the correct localisation of proteins is crucial for their correct processing and function. Moreover, proteins and the cellular processes they partake in are regulated by relocalisation in response to various cellular stimuli. High-throughput ‘omics experiments result in a list of proteins or genes of interest; one way in which their functional role can be understood is through the knowledge of their subcellular localisation, as deduced through statistical enrichment for Gene Ontology Cellular Component (GOCC) annotations or similar. We have designed a bioinformatics tool, named SubcellulaRVis, that compellingly visualises the results of GOCC enrichment for quick interpretation of the localisation of a group of proteins (rather than single proteins). We demonstrate that SubcellulaRVis precisely describes the subcellular localisation of gene lists whose locations have been previously ascertained. SubcellulaRVis can be accessed via the web (http://phenome.manchester.ac.uk/subcellular/) or as a stand-alone app (https://github.com/JoWatson2011/subcellularvis). SubcellulaRVis will be useful for experimental biologists with limited bioinformatics expertise who want to analyse data related to protein (re)localisation and location-specific modules within the intracellular protein network.

## Graphical Abstract

**Graphical Abstract:**
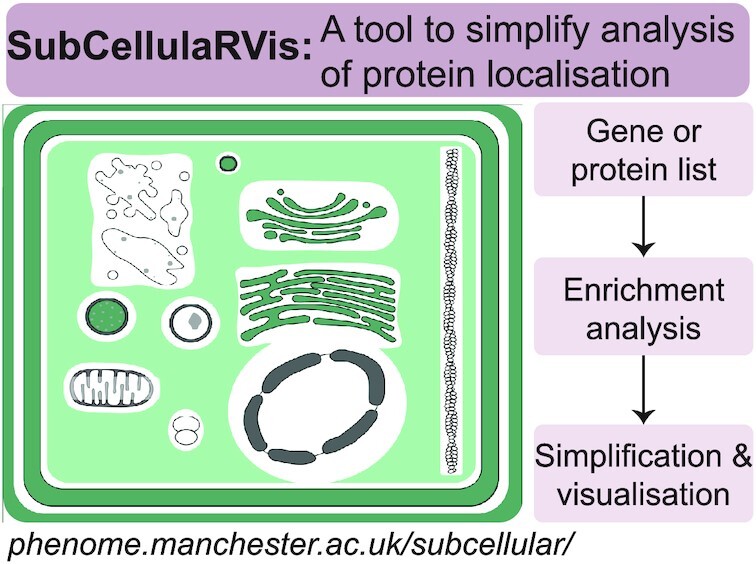
SubcellulaRVis is a user-friendly tool to simplify and visualise enrichment of Gene Ontology (GO) cellular compartment terms in omics datasets, aiding understanding of protein localisation.

## INTRODUCTION

The localisation of proteins within cells is critical for their processing, function and regulation. Cellular homeostasis and response to environmental signals are also dependent on sequestering or dynamic re-localisation of proteins in specific intracellular compartments (1). Regardless of whether a protein is relocated in the cell or remains in a single compartment for the duration of its life, spatial regulation is critical for protein function. For example, upon translation, proteins bound for secretion will be trafficked through multiple compartments that compose the endomembrane system; contained within these compartments are ‘quality control’ proteins that mediate correct folding or degradation for malformed proteins (2). Cellular processes also occur in discrete, non-membrane bound locations, such as degradation within the proteasome, or transcriptional regulation in stress granules (3,4). It is also increasingly appreciated that proteins which are dynamically recruited to different subcellular locations form functionally active modules that may be location specific, such as those that form and disperse at the lysosomal surface in response to growth factor or nutrient sensing (5). Moreover, proteins can perform different functions dependent on their locations – referred to as moonlighting proteins (6,7). The change in protein localisation introduces them to different protein partners that may differentially regulate the function of the protein, as exemplified by STAT3, which has independent roles in transcriptional regulation and oxidative phosphorylation (8). Proteins can also be pathologically mislocated, as demonstrated by mislocalisation of liver-specific peroxisomal enzyme alanine:glyoxylate aminotransferase (AGT) to the mitochondria in patients diagnosed with primary hyperoxaluria type 1 (9).

Co-regulated proteins or genes are commonly identified using high-throughput ‘omics experiments, such as those based on transcriptomics, proteomics and phosphoproteomics. The regulated proteins or genes identified in these experiments are often assessed based on enrichment for annotations to particular biological characteristics or participation in biological pathways. Annotations based on subcellular localisation are stored in the Gene Ontology aspect Cellular Component (GOCC) or the Jensen COMPARTMENTS database (10,11). Enrichment for these annotations can provide initial indications of location-specific roles for a protein or gene list. Moreover, when analysing spatially resolved data, annotations describing subcellular localisation also provide an important data quality control. This is exemplified in the analysis of spatial proteomics data that utilise proximity dependent biotinylation, in which bait proteins (known, characterised proteins tagged with a biotin ligase or peroxidase) covalently modify neighbouring proteins (termed ‘prey’) through the addition of biotin (12,13). Isolating the biotinylated proteins generates a spatial interactome of the bait protein. Recently, BioID-based proximity-dependent biotinylation was used to generate cell-wide maps of proteins localised to particular organelles (14). As demonstrated in this work, calculating enrichment for GOCC terms is a useful method for understanding whether the bait has been correctly tagged, in which case one would expect enrichment for the subcellular compartment the bait is commonly found in.

Several web-based tools, such as EnrichR (15), can be used to perform enrichment analyses on protein or gene lists based on the GOCC or compartments databases. These analyses are accessible to biologists with minimal bioinformatics skills and are useful for understanding the subcellular compartments overrepresented in a list of proteins or genes of interest. However, a significant barrier in the interpretation of the results is that annotations are often highly specific, meaning it can be difficult to extract general trends from the potentially long list of returned annotations. For example, the enrichment for GOCC terms,calculated using EnrichR, on a list of GPI-anchored proteins (extracted from UniProt, Supplementary Table S1), results in a list of terms that are difficult to immediately generalise (Table 1), despite useful visualisation tools included in the EnrichR web app. In contrast, certain cellular compartments are poorly characterised, for example the individual compartments of the endosomal system (16), and therefore analyses of proteins localised in such compartments may be noisy and incomplete.

**Table 1.** Top 10 GOCC terms from results of GOCC enrichment, calculated using EnrichR on a list of 139 GPI-anchored proteins

GOCC Term	Overlap	*P*-value	Adjusted *P*-value
anchored component of plasma membrane (GO:0046658)	33/46	3.16E-63	2.31E-61
intrinsic component of external side of plasma membrane (GO:0031233)	17/24	1.46E-32	5.35E-31
anchored component of external side of plasma membrane (GO:0031362)	16/20	3.50E-32	8.52E-31
membrane raft (GO:0045121)	15/163	3.20E-13	5.84E-12
secretory granule membrane (GO:0030667)	16/274	5.34E-11	7.79E-10
Golgi lumen (GO:0005796)	8/100	3.86E-07	4.70E-06
lysosomal lumen (GO:0043202)	7/86	1.88E-06	1.96E-05
specific granule membrane (GO:0035579)	6/91	3.52E-05	3.22E-04
vacuolar lumen (GO:0005775)	7/161	1.12E-04	9.11E-04
plasma membrane raft (GO:0044853)	5/82	2.34E-04	0.001708

**Figure 1.**
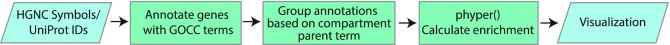
SubcellulaRVis workflow.

We have implemented a Shiny web app, named SubcellulaRVis, that provides graphical visualisation of subcellular compartments enrichment from a list of multiple genes or proteins. By performing the enrichment test using condensed annotations, so that there is a single term for each major intracellular compartment in the results, the analyses can be concisely visualised and interpreted. SubcellulaRVis represents an alternative to the visualisation provided on the web interfaces of UniProt and COMPARTMENTS which only visualise the localisation of a single protein (11,17). A major novelty of our approach is the interactive visualisation on a schematic of the eukaryotic cell of GOCC enrichment (Figure 1), allowing for user-friendly interpretation, exploration, and presentation of the data. The visualisation can be exported as a static image or as a table of the data behind the visualisation. SubcellulaRVis provides a solution for non-bioinformaticians to investigate the subcellular localisation of the proteins within a dataset of interest and standardises the visualisation of these results. The app is available at: http://phenome.manchester.ac.uk/subcellular/.

## MATERIALS AND METHODS

SubcellulaRVis aims to simplify the interpretation of GOCC enrichment analyses through visualisation of a standardised schematic of the cell. We selected subcellular compartments that are non-transient and common to eukaryotes, though it is a non-exhaustive list.

**Figure 2.**
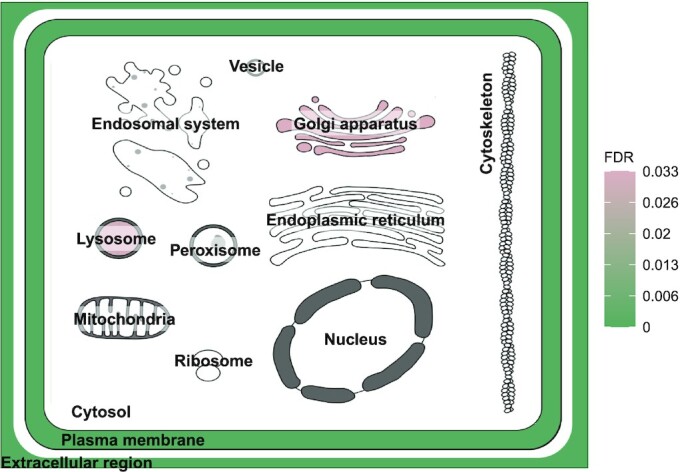
Visualisation of enrichment results using SubcellulaRVis on a gene list of GPI anchored proteins (Supplementary Table S1).

We first defined each cellular compartment visualised on the schematic (as shown in Figure 2) in respect to the GOCC terms that describe it. We did this by utilising the hierarchical structure of GO. The GO terms are hierarchically organised, with GO terms towards the ‘top’ of the hierarchy being more general descriptors (e.g. GO:0005886, plasma membrane.) whilst terms towards the ‘bottom’ of the hierarchy are more specific (e.g. GO:0005901, caveola) (18). Each term in the GO hierarchy has parent terms (those that are higher in the hierarchy) and may also have child terms (those that are lower in the hierarchy). We identified the highest level GOCC term that would best describe each of the compartments visualised on the schematic (Table 2). Then, using the GO.db package in Bioconductor (version 3.14) (19), the child terms of the high-level GOCC terms were extracted and associated together. We call these grouped, compartment-specific terms the SubcellulaRVis compartment and use the high-level parent term to describe them, as shown in Table 1, hereafter. We extracted all the genes annotated to the terms in each SubcellulaRVis compartment using the species related AnnotationData libraries in Bioconductor (version 3.14), to create gene sets against which to calculate enrichment (Table 2). The annotation lists were generated for multiple species: *H. sapiens, M. musculus, D. melanogaster, S. cerevisiae, R. rattus* and *X. laevis*.

**Table 2.** Number of child terms for each parent term selected

Parent compartment	GOID	Number of child terms	Number of genes annotated					
			*H. sapiens*	*M. musculus*	*D. melanogaster*	*S. cerevisiae*	*R. rattus*	*X. laevis*
Cytoplasm	GO:0005737	1230	11 847	11 262	4700	4083	10 436	4884
Cytoskeleton	GO:0005856	273	2302	2213	523	255	2111	889
Endoplasmic reticulum	GO:0005783	110	1961	1777	499	635	1700	685
Endosome	GO:0005768	77	978	906	240	189	846	296
Extracellular region	GO:0005576	94	4570	2697	1036	110	2072	1102
Golgi apparatus	GO:0005794	47	1608	1468	379	285	1398	622
Intracellular vesicle	GO:0097708	294	2405	1965	423	297	1848	555
Lysosome	GO:0005764	38	697	472	106	6	456	145
Mitochondrion	GO:0005739	96	1627	1852	863	1129	1570	650
Nucleus	GO:0005634	492	8072	7127	2827	2302	6585	3673
Peroxisome	GO:0005777	19	136	146	98	88	149	66
Plasma membrane	GO:0005886	471	5726	5343	1383	507	5779	1878
Ribosome	GO:0005840	20	243	231	285	267	295	239

Enrichment is calculated on the user supplied list of HUGO Gene Nomenclature Committee (HGNC) symbols or UniProt IDs (Figure 1). Firstly, duplicate and non-valid entries are removed from the list. Then, the genes are annotated to the GOCC terms based on the annotation lists generated for each species, and the annotations are then grouped based on the SubcellulaRVis compartment. A standard enrichment test is performed using the hypergeometric probability function, *phyper*, in R (version 4.1.0), to calculate the enrichment for each SubcellulaRVis compartment in the user-supplied gene list, and corrected using the false discovery rate (FDR) using the *p.adjust* function in R with the ‘method’ argument set to ‘fdr’. Though it has limitations (20,21), the hypergeometric test was selected as values associated to the genes or proteins (e.g. expression or abundance values) do not need to be supplied in order to perform the test. To improve the accuracy of this calculation, the user can also input a background population of expressed genes or proteins in their sample, if known (as discussed in (22)). In the absence of the user-supplied background population list, the calculation is performed based on all the genes in the reference genomes (from the Bioconductor AnnotationData databases, as described above).

The SubcellulaRVis tool has been implemented as a Shiny (version 1.7.1) web app (http://phenome.manchester.ac.uk/subcellular/) and as a standalone app with R package (https://github.com/JoWatson2011/subcellularvis). The R package will be submitted to Bioconductor upon acceptance of the article. The visualisation within the app has been implemented using ggplot2 (version 1.7.1) and Plotly (version 4.10.0), allowing interactive exploration of the results when viewed on a web browser. All dependencies are described in the DESCRIPTION file of the R package and will be installed along with the package, as described in the README file.

## RESULTS

### Features of SubcellulaRVis

The key feature of the SubcellulaRVis app is the visualisation of GOCC enrichment results on a graphical representation of a eukaryotic cell (as seen in Figure 2), allowing for rapid and simple interpretation of the predominant localisation of proteins of interest. By providing visualisation for a group of proteins, we provide an alternative to similar tools that visualise the localisation of single proteins (11,17). The app provides an interactive view of protein localisation, and a static image of the plot can be exported; the characteristics of both the image and the plot (such as text size or colour scale) can be chosen by the user. This is demonstrated by the test data, available in the app, which is a list of 139 proteins which are annotated as GPI-anchored in UniProt (Supplementary Table S1) (17). Figure 2 visualises the enrichment of these proteins for the membrane bound organelles of the secretory pathway, plasma membrane and for the extracellular space (Table 3). Cellular compartments in white are not enriched for in the gene list.

**Table 3.** Enrichment results using SubcellulaRVis for gene list of 139 GPI-anchored proteins. Break in table indicates non-significant enrichment

Compartment	*P*-value	FDR	Number of genes in user list annotated to compartment
Plasma membrane	1.36E-71	1.91E-70	137
Extracellular region	1.08E-47	1.40E-46	114
Lysosome	0.002651275	0.031815304	12
Golgi apparatus	0.002986299	0.032849289	21
Vacuole	0.007331236	0.073312358	12
Intracellular vesicle	0.011034454	0.099310088	27
Endoplasmic reticulum	0.058046176	0.464369405	20
Cytoplasm	0.999999569	1	59
Nucleus	1	1	10
Cytoskeleton	0.999999789	1	1
Mitochondrion	0.999957536	1	1
Ribosome	0.833831999	1	0
Endosome	0.450305359	1	7
Peroxisome	0.654277871	1	0

Using SubcellulaRVis, the user can view the results of the enrichment analysis graphically, as discussed above, or in tabular form. A standard enrichment including all the GOCC terms (rather than the summarised terms) can be calculated and can be viewed in a separate tab on the app; the table also specifies the SubcellulaRVis compartment each of these terms is assigned to, allowing the user full understanding of the way their data has been analysed and visualised. To evaluate genes or proteins that have been annotated to multiple categories, the user can use the results found in the ‘overlap’ tab, which contains an UpSet plot and the results in tabular form. Finally, we have also implemented a feature to allow users to compare multiple gene lists. We anticipate this being useful to users who have multiple experimental conditions or want to quickly perform and visualise multiple comparisons.

### Validation of GO cellular compartment summarisation

To assess the precision of the SubcellulaRVis compartment assignments, we validated the tool using various data sets describing subcellular localisation of proteins.

The first validation data set came from The Human Protein Atlas (23). The subcellular section of the database is considered a gold-standard resource for the assignment of protein localisation. Each entry in the database is a protein whose spatial distribution has been investigated with immunofluorescence, confocal microscopy and proteomics in up to three different cell lines (23). The flat file of subcellular location data was downloaded from the website (https://www.proteinatlas.org/about/download), filtered for proteins that were associated to only one subcellular compartment and protein lists were created associated to each subcellular compartment. Analysis of proteins with limited spatial distribution allowed for simpler validation, as we could perform a one-to-one comparison between the true localisation and the highest enriched SubcellulaRVis compartment. We found that this was indeed the case for many of the protein lists (Table 4, Supplementary Table S2). Encouragingly, sub-compartments were resolved to their parent compartments; for example, proteins associated to actin filaments, intermediate filaments and microtubules were all associated to the cytoskeleton. Likewise, cell junctions and focal adhesion sites were associated to the plasma membrane. Moreover, the parent compartments were correctly associated, for example the endoplasmic reticulum and mitochondria. Only the peroxisome proteins were not correctly associated by the most enriched compartment however the second most enriched compartment was the peroxisome (Supplementary Table S2); this is due to overlap between the genes annotated to the peroxisome and the cytoplasm.

**Table 4.** SubcellulaRVis enrichment results for each of the protein lists associated to cellular compartments in the Human Protein Atlas database (23). The final column represents the overlap between the user-supplied gene list and genes annotated to the highest enriched SubcellulaRVis compartment

Experimentally determined cellular compartment	Highest enriched SubcellulaRVis compartment	FDR	No. user genes annotated to comp./no. user genes
Actin filaments	Cytoskeleton	7.11999E-07	14/29
Cell Junctions	Plasma membrane	1.0576E-06	32/50
Centriolar satellite	Cytoskeleton	0.016808022	7/25
Centrosome	Cytoskeleton	4.1902E-17	37/78
Cytoplasmic bodies	Cytoplasm	0.097245905	10/11
Cytosol	Cytoplasm	1.07583E-64	732/932
Endoplasmic reticulum	Endoplasmic reticulum	2.8002E-109	158/234
Focal adhesion sites	Plasma membrane	0.260811294	8/18
Golgi apparatus	Golgi apparatus	1.08243E-68	122/262
Intermediate filaments	Cytoskeleton	6.62768E-14	26/50
Lipid droplets	Endoplasmic reticulum	0.000474586	6/12
Microtubules	Cytoskeleton	8.88401E-09	22/51
Mitochondria	Mitochondrion	< 2.23e-308	399/557
Nuclear bodies	Nucleus	2.88368E-09	47/72
Nuclear membrane	Nucleus	0.008171852	23/37
Nuclear speckles	Nucleus	1.08505E-40	157/188
Nucleoli	Nucleus	2.62163E-12	63/84
Nucleoli fibrillar center	Nucleus	0.016402093	23/42
Nucleoli rim	Nucleus	< 2.23e-308	6/7
Nucleoplasm	Nucleus	< 2.23e-308	1357/1666
Peroxisomes	Cytoplasm	< 2.23e-308	14/14
Plasma membrane	Plasma membrane	1.91615E-76	242/317
Vesicles	Intracellular vesicle	2.1049E-42	191/594

We then validated SubcellulaRVis with two experimental datasets. The first, from Go *et al.* (14) (Supplementary Table S8 of original publication), used proximity dependent biotinylation to label proteins in the vicinity of different organellar markers which were tagged with the biotin ligase enzyme BioID. In total, 4424 proteins were identified and associated to different subcellular compartments. We calculated the enrichment using SubcellulaRVis of the proteins associated to the different organelles in the datasets, treating them as organelle-specific protein lists. The most significantly enriched SubcellulaRVis compartment for each of the twenty experimentally determined, organelle-associated protein lists were directly or closely matched (Table 5, Supplementary Table S3). For example, proteins experimentally associated to the cell junction, nucleolus and microtubules were enriched for the plasma membrane, nucleus, and cytoskeleton compartments, respectively by the SubcellulaRVis tool. This case study provides a useful example of how analysis based on standardised, generic cellular compartments names, such as the SubcellulaRVis compartments, could be useful for comparison between different spatial studies.

**Table 5.** SubcellulaRVis enrichment results for each of the protein lists associated to cellular compartments by Go *et al.* (14). The final column represents the overlap between the user-supplied gene list and genes annotated to the highest enriched SubcellulaRVis compartment

Experimentally determined cellular compartment	Highest enriched SubcellulaRVis compartment	FDR	No. user genes annotated to comp./no. user genes
Cell junction	Plasma membrane	5.8676E-35	153/235
Chromatin	Nucleus	2.3261E-126	354/383
ER membrane	Endoplasmic reticulum	4.93713E-58	86/127
Mitochondrial matrix	Mitochondrion	4.6055E-262	266/299
Actin, cytosol	Cytoskeleton	2.74859E-57	142/300
ER lumen	Endoplasmic reticulum	7.10322E-88	122/165
Endosome, lysosome	Endosome	4.74921E-68	87/173
Nucleolus	Nucleus	5.18092E-48	145/164
Nucleoplasm	Nucleus	9.37741E-66	222/247
Nuclear body	Nucleus	1.1701E-102	279/295
Plasma membrane	Plasma membrane	1.21931E-52	248/376
Centrosome	Cytoskeleton	5.82532E-58	130/258
Mitochondrial Membrane, Peroxisome	Mitochondrion	1.41277E-32	74/189
Golgi	Golgi apparatus	2.10532E-62	89/146
Nuclear outer membrane-ER membrane network	Endoplasmic reticulum	7.25607E-97	159/262
Cytoplasmic RNP granule	Cytoplasm	5.44657E-12	120/143
Microtubules	Cytoskeleton	1.68917E-59	87/115
Mitochondrial inner membrane, Mitochondrial inter membrane space	Mitochondrion	1.9238E-99	106/122
Misc.	Cytoplasm	4.80374E-22	237/279
Early endosome, Recycling endosome	Intracellular vesicle	6.73788E-37	80/146

To assess the precision of the SubcellulaRVis tool for the analysis of a non-human experimental data set we extracted the data from Nightingale et al. (24) (from Supplementary Table S3 of original publication) which used the hyperplexed Localisation of Organelle Proteins by Isotope Tagging (hyperLOPIT) method (25) to reconstruct spatial profiles of organelle proteomes in *Saccharomyces cerevisiae*. SubcellulaRVis found the highest enrichment for the compartment most closely describing the experimentally associated cellular compartment for the cytosol, nucleus, Golgi apparatus and plasma membrane (Table 6) and the second highest enriched compartment for the mitochondrion and endoplasmic reticulum (Supplementary Table S4). The latter is explained by the significant overlap in genes annotated to the cytoplasm and associated to the mitochondria (183/186) and endoplasmic reticulum (165/170) by the hyperLOPIT experiment. We concluded that SubcellulaRVis accurately replicates the protein localisation determined in *S. cerevisiae*. Moreover, the SubcellulaRVis visualisation (as demonstrated in Figure 1) would be an impactful way to display the enrichment results for each of the organelle-specific lists.

**Table 6.** SubcellulaRVis enrichment results for each of the protein lists associated to cellular compartments by Nightingale *et al.* (24). The final column represents the overlap between the user-supplied gene list and genes annotated to the highest enriched SubcellulaRVis compartment

Experimentally determined cellular compartment	Highest enriched SubcellulaRVis compartment	FDR	No. user genes annotated to comp./no. user genes
Cytosol	Cytoplasm	9.75327E-27	743/894
ER	Cytoplasm	<2.23e-308	165/170
Golgi	Golgi apparatus	1.60496E-86	80/109
Mitochondrion	Cytoplasm	<2.23e-308	183/186
Nucleus	Nucleus	1.98944E-35	533/865
Plasma membrane	Plasma membrane	1.32569E-69	160/401

## DISCUSSION

We have introduced an app named SubcellulaRVis, which provides easy visualisation of the enriched subcellular locations from a user-supplied list of HGNC symbols or UniProt IDs. The visualisation is more biologically insightful than a bar chart and will aid in efficient interpretation of the characteristics of gene lists and standardisation of subcellular localisation analyses. Moreover, SubcellulaRVis complements existing visual tools such as COMPARTMENTS and UniProt which use similar schematics to visualise the known locations of individual protein species. We anticipate our software will be particularly useful for cellular and molecular biologists with limited bioinformatics expertise wanting to perform precise and quick enrichment analysis and immediate visualisation of gene lists. SubcellulaRVis will be beneficial in analysing data from spatial proteomics, as we have demonstrated here, which is increasingly used to resolve organelle proteomes and location-specific protein interaction networks. In this context, our tool will provide impactful visualisation of how protein localisation varies upon different extracellular perturbations or for providing confirmation of correct tagging of proteins for spatial proteomics experiments. Moreover, the method is useful for analysing high-throughput data from all ‘omics contexts. For example, RNA transcripts can be localised in order to be translated at the location in which the protein will be active (26). This information is captured in the database RNAlocate but, as the authors note, RNA localisation is not well-captured in the Gene Ontology resource (on which our tool is based) (27). Therefore, SubcellulaRVis is well suited for analysing protein lists or protein products from genes or transcript lists.

**Figure 3.**
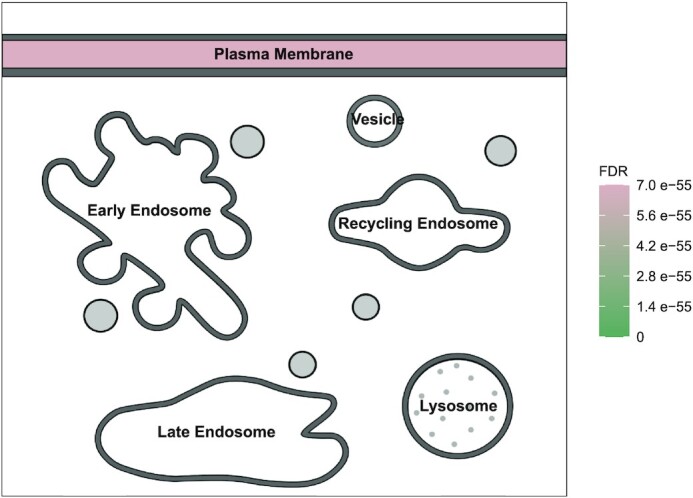
Visualisation of enrichment results using SubcellulaRVis, using the trafficking-specific endosomal view, on a gene list of GPI anchored proteins (Supplementary Table S1).

There are some features of SubcellulaRVis that will be developed further based on increasing and more resolved information on the subcellular localisation of proteins. We have included the option to calculate enrichment within human protein or gene lists based on predicted subcellular localisations from the Human Protein Atlas rather than GOCC annotations. At the time of writing, the subcellular map of the Human Protein Atlas has coverage of 65% of human protein-coding genes. This makes it an invaluable resource for researchers and a good fit for SubcellulaRVis. SubcellulaRVis also provides an alternative view for the visualisation of the endosomal system (Figure 3). The endosomal system is notoriously hard to characterise (16), therefore our tool will be a valuable resource for distinguishing gene lists based on enrichment for different endosomal compartments. The endosomal view of SubcellulaRVis has recently been effectively utilised for visualisation of spatially resolved phosphoproteomics data describing intracellular signalling modules following dynamic cell surface receptor internalisation (28). In the future, SubcellulaRVis can be further expanded to provide more detailed views of sub-compartments of, for example, the nucleus or endomembrane systems.

In summary, we have described an easy-to-use and powerful tool for the visualisation and simplification of GOCC enrichment analyses on gene or protein lists.

## DATA AVAILABILITY

The SubcellulaRVis web app is hosted at http://phenome.manchester.ac.uk/subcellular/ and the software can be installed at https://github.com/JoWatson2011/subcellularvis.

## Supplementary Material

gkac336_Supplemental_Files
